# ER stress and viral defense: Advances and future perspectives on plant unfolded protein response in pathogenesis

**DOI:** 10.1016/j.jbc.2025.108354

**Published:** 2025-02-25

**Authors:** Binita Adhikari, Jeanmarie Verchot, Federica Brandizzi, Dae Kwan Ko

**Affiliations:** 1 Department of Plant Pathology and Microbiology, Texas A&M University, College Station, Texas, USA; 2 MSU-DOE Plant Research Lab, Michigan State University, East Lansing, Michigan, USA; 3 Great Lakes Bioenergy Research Center, Michigan State University, East Lansing, Michigan, USA; 4 Department of Plant Biology, Michigan State University, East Lansing, Michigan, USA

**Keywords:** unfolded protein response, viral infections, protein homeostasis, ER stress, antiviral defense, potexvirus

## Abstract

Viral infections pose significant threats to crop productivity and agricultural sustainability. The frequency and severity of these infections are increasing, and pathogens are evolving rapidly under the influence of climate change. This underscores the importance of exploring the fundamental mechanisms by which plants defend themselves against dynamic viral threats. One such mechanism is the unfolded protein response (UPR), which is activated when the protein folding demand exceeds the capacity of the endoplasmic reticulum, particularly under adverse environmental conditions. While the key regulators of the UPR in response to viral infections have been identified, our understanding of how they modulate the UPR to suppress plant viral infections at the molecular and genetic levels is still in its infancy. Recent findings have shown that, in response to plant viral infections, the UPR swiftly reprograms transcriptional changes to support cellular, metabolic, and physiological processes associated with cell viability. However, the underlying mechanisms and functional outcomes of these changes remain largely unexplored. Here, we highlight recent advances in plant UPR research and summarize key findings related to viral infection–induced UPR, focusing on the balance between prosurvival and prodeath strategies. We also discuss the potential of systems-level approaches to uncover the full extent of the functional link between the UPR and plant responses to viral infections.

Plant virus outbreaks pose significant threats to crop growth, development, and environmental sustainability, resulting in substantial global reductions in productivity (1, 2). These challenges are exacerbated by climate change, as higher temperatures and reduced water availability not only compromise plant productivity but also create conditions conducive to the emergence of new pathogenic virus strains (3). Consequently, there is an urgent need to advance our understanding of host genetic defenses against plant viruses, which can be integrated into breeding programs to develop resilient crops. Failure to address these needs will profoundly impact where and how crops are grown, with far-reaching consequences for regional and global economies. Plants and other eukaryotes have evolved sophisticated mechanisms to manage protein synthesis, folding, and maturation in the endoplasmic reticulum (ER), a process critical for cellular function (4). The demand for protein synthesis in the ER fluctuates in response to genetic programs controlling growth and development as well as environmental stressors (5, 6, 7). Plant RNA viruses, in particular, impose significant demands on the ER by increasing protein synthesis and trafficking in the ER lumen, often exceeding its capacity (8, 9, 10). This overload can result in the accumulation of misfolded proteins, a condition known as ER stress, which activates the unfolded protein response (UPR), a set of signaling pathways aimed at restoring ER homeostasis (11, 12). The UPR is intricately linked to the heightened demand for *de novo* synthesis of immunity-related proteins during viral infections (13). This is partially achieved through signaling pathways that swiftly reprogram the expression of genes encoding ER-resident enzymes and chaperones, enhancing the ER's folding capacity to cope with the viral demands (9, 10). Concurrently, proteins within the ER may be selectively degraded through the ER-associated protein degradation (ERAD) or ER-phagy machinery (14, 15). Together, these molecular mechanisms create a complex network of genetic, transcriptional, and post-transcriptional responses at the intersection of viral infection, plant immunity, and development. Deciphering this network offers significant potential for improving the immune resilience of crops.

Recent studies have shed new light on the proviral and antiviral roles of the UPR and ERAD machinery in plants. Early studies involving potexvirus gene block 3 (TGB3), a transmembrane ER-associated protein, indicated that its overaccumulation can be cytotoxic and its removal by ERAD coordinated the UPR activation, alleviated ER stress, and thus provided protection against cell death (16, 17, 18). However, a wider number of virus species have since been shown to engage with UPR sensor proteins, including basic leucine zipper 28 (bZIP28), bZIP17, the inositol-requiring enzyme (IRE1), along with IRE1's downstream effector bZIP60 (16, 19, 20). These functional interactions play crucial roles in promoting or limiting viral infections beyond encouraging the cell to manage the demand for increasing levels of viral protein synthesis. We have also learned that the UPR signaling pathways can be stimulated by viruses that reshape ER membranes into cellular centers housing the viral replication and assembly machinery. Finally, there are new reports of viruses encoding proteins that can block key components of the UPR machinery in a manner that resembles an arms race between the virus and the host immune system (8, 19, 21).

Over the past decade, our understanding of how the plant UPR machinery engages with pathogen infections, especially viral infection, and the functional consequences has greatly advanced because of genetic, genomic, and technological innovations as well as the accumulation of new knowledge of plant UPR. In this review, we provide a comprehensive overview of plant UPR signaling highlighting recent advances that reveal how it is tuned to orchestrate interconnected signaling pathways, thus operating as a moderator to control cell fate. Next, we examine the recent advances in virus interactions with the UPR and present new models based on disparate virus interactions with the ER. We also emphasize the importance and application of new research innovations including a systems-level understanding of the governing role of the UPR in response to viral infections. Finally, we discuss emerging questions and technological advancements that could expand our understanding of plant UPR in infections caused by viruses and other pathogens.

## The underlying mechanisms of plant UPR

While the UPR is found across eukaryotic organisms, it appears to have evolved with kingdom-specific modifications at its core (22, 23, 24, 25). For example, while mammals have the three distinct, yet partially redundant, regulatory branches (IRE1, activating transcription factor 6 [ATF6], and PKR-like endoplasmic reticulum kinase [PERK]), yeast (*Saccharomyces cerevisiae*) has only IRE1 and plants have IRE1 and bZIP28, lacking the counterpart of PERK (11, 22). Under physiological conditions, the luminal domains of IRE1 or bZIP28 are physically associated with binding immunoglobulin proteins (BiPs) *via* the ER-luminal sensing domain, thus being inactive (26). Due to their higher affinity for misfolded proteins than for these UPR sensors, the accumulation of misfolded proteins leads to the disassociation of BiPs from the ER stress sensor proteins, which are then activated to transduce the intracellular ER stress signaling. In addition to the core factors, the UPR consists of other nonessential, yet critical, regulators for cellular viability. In this section, we discuss the most updated models of the mechanisms underlying the UPR regulation in plants. For extensive discussion regarding plant UPR, its underlying mechanisms, and biological significance, we refer the readers to other reviews (12, 22, 23).

### The highly conserved IRE1 branch of the UPR

IRE1, an ER-resident type I transmembrane dual-functioning kinase/endoribonuclease, stands out as the most evolutionarily conserved stress transducer of the UPR. Unlike in yeast, paralogs of IRE1 exist in mammals and plants, resulting from gene duplication events across evolution (27, 28). In mammals, IRE1α has a predominant role over IRE1β (29). Its RNase activity splices *XBP1* mRNA to produce the nucleus-localized XBP1 transcription factor (TF). IRE1 engages with TRAF2 to provoke the c-Jun N-terminal kinase signaling leading to apoptosis (30). Investigations of the *Arabidopsis thaliana* (hereafter, Arabidopsis) IRE1a and IRE1b show that they are functionally redundant in their ability to unconventionally splice *bZIP60* (the counterpart of *XBP1*) mRNA, creating a frameshift in a way that the spliced bZIP60 (sbZIP60) TF contains a nuclear localization signal (31, 32). The newly discovered Arabidopsis IRE1c is not essential for the UPR but plays a role in normal physiological conditions (33, 34). Upon the splicing event by IRE1, sbZIP60 translocates into the nucleus and simultaneously regulates the expression of genes associated with numerous biological processes *via* ER stress–responsive *cis*-regulatory elements (CREs) in highly interconnected networks in concert with bZIP28 (35, 36). IRE1 also carries out RNase activity in a process known as regulated IRE1-dependent decay (RIDD), for cleaving RNAs (mRNAs, rRNA, and miRNA) to suppress translation or induce critical cellular programs (37, 38). Intriguingly, the specificity of RIDD remains unclear: a consensus sequence for RIDD substrates within a stem-loop motif is lacking in other targets (39). RIDD is less investigated particularly in plants, partly because of the technical difficulty in handling plant tissues (*e.g.*, nuclei isolation for global run-on sequencing (40) or drug penetrance into plant cell wall for transcriptional inhibition (38)). Nonetheless, a few studies have revealed that RIDD targets are highly enriched for numerous cellular responses associated with the secretory pathway (38, 41), suggesting that failure to degrade those mRNAs could cause both an excessive burden of protein folding at the early stage of ER stress and cell death under prolonged ER stress, respectively. In yeast, deletion of *IRE1* (*ire1Δ*) eliminated the transcriptional induction of critical ERAD-related genes under ER stress (42, 43), indicating that the UPR regulates ERAD through IRE1. A model whereby IRE1-XBP1/bZIP60 signaling regulates the expression of genes required for ERAD is supported by studies of mammalian viruses such as hepatitis virus C and other flaviviruses (14, 15). There is limited evidence in plants during viral infection, and conclusive genetic studies have not yet been performed.

A recent study of the *IRE1* gene family across 90 diverse plant species highlights copy number variations of *IRE1* genes in plants (44). A significant number of monocot plant species have a single *IRE1* gene (*e.g.*, barley [*Hordeum vulgare*], rice [*Oryza* ssp.], and sorghum [*Sorghum bicolor*]), although maize (*Zea mays*) has two and wheat (*Triticum aestivum*) has three *IRE1* genes. Among dicotyledonous plants, the numbers of *IRE1* genes can vary between 2 and 11. While Arabidopsis has three genes (*IRE1a*, *IRE1b*, and *IRE1c*) as described previously, rapeseed (*Brassica napus*), camelina (*Camelina sativa*), and rose gum (*Eucalyptus grandis*) have six, ten, and 11 *IRE1* genes, respectively. The wide-ranging number of *IRE1* copies across eudicot species points to an evolutionary expansion of the UPR machinery, which has been underexplored, partly because of the lack of technology to perform reverse genetic studies in nonmodel plant species. Considering the plant taxa that have between three and 11 *IRE1* genes, and current theories that all *IRE1s* are functionally redundant and arose by gene duplication, one has to assume that there must be an expansion of gene function with the expansion in the size of gene family members. One explanation is that *IRE1s* are vulnerable to inactivation, and the expansion of the *IRE1* gene family is a defensive strategy to maintain the integrity of the UPR machinery. Another possibility is that gene promoters modulate tissue specificity and developmental controls in a manner that necessitates gene expansion for rapid UPR activation in response to development and environmental stress.

### The bZIP28-mediated branch of the UPR

Serving as a multiphasic network web of signaling pathways to cope with ER stress, plant UPR is also transduced by bZIP28, a type II transmembrane protein. Following the disassociation from BiPs, bZIP28 moves to the Golgi apparatus where it is progressively cleaved by as-yet unidentified protease (*i.e.*, not by site-1 protease) and site-2 protease, releasing a fragment containing the bZIP domain (45). The activated form of bZIP28 translocates to the nucleus where it regulates transcriptional changes required for ER homeostasis in a concerted manner with sbZIP60 *via* ER stress–responsive CREs including ER stress–responsive element-I (ERSE-I; 5′-CCAAT-N_10_-CACG-3′) and unfolded protein response element-I (5′-TGACGTG-G/A-3′) (35, 46, 47, 48). Gene regulation studies have revealed interesting features of bZIP28 and bZIP60 in the UPR. First, they are functionally redundant under ER stress as demonstrated by no pronounced phenotype of single mutants of each gene while their double mutants are completely lethal under ER stress. Second, bZIP28 and bZIP60 are partially redundant in recovery from ER stress. For example, *bzip28-2*, a knockout mutant of *bZIP28*, showed a slower recovery from ER stress compared with Col-0 and *bzip60-2*, a knockout mutant of *bZIP60*, which exhibited a delayed recovery relative to Col-0 (35, 49). Gene regulation analyses revealed hundreds of genes, of which expression depends on either bZIP28 or bZIP60 during ER stress recovery (35). In addition, in the same study, *de novo* motif analyses on the promoters of those genes revealed CREs that could be bound by other TFs, suggesting an involvement of other TFs in the regulatory dynamics of UPR-bZIP TFs.

### Other ER membrane–anchored proteins in the plant UPR

Although IRE1 and bZIP28 are core ER stress sensors anchored in the ER membrane that amplify stress signals to drive essential molecular responses, other ER membrane–anchored proteins, while not essential, play critical roles in stress tolerance and cell survival. One such protein is bZIP17, an ER membrane sensor activated by sequential proteolytic cleavage, similar to bZIP28. Interestingly, while bZIP17 has been shown to physically interact with bZIP28 or bZIP60 in yeast, this interaction has not been demonstrated in plants or *in vitro* (46). Although bZIP17 is involved in physiological development and response to salt stress (50, 51), its genetic perturbation minimally impacts gene reprogramming under ER stress (36), indicating that it is not a primary regulator in the UPR. Under heat stress, which often intersects with ER stress, plant-specific NAC (no apical meristem, Arabidopsis transcription activation factor and Cup-shaped cotyledon) TFs NAC089 and NAC062, along with B-cell lymphoma 2–associated athanogene 7 (BAG7), are proteolytically processed from their membrane-anchored forms (ER or plasma membrane) to translocate to the nucleus, where they drive extensive transcriptional reprogramming essential for stress tolerance (52, 53, 54). The ER stress–inducible expression of *NAC089* and *NAC062* depends on bZIP28 and/or bZIP60, and both factors promote programmed cell death (PCD) by activating PCD-related genes. In contrast, BAG7 responds to heat shock–induced ER stress by coordinating transcriptional changes that enhance stress tolerance. In addition, asparagine-rich protein (NRP1) and NRP2, homologous proteins anchored to the ER, are transcriptionally induced by bZIP60 during ER stress (55). These proteins inhibit cell death, although the underlying mechanisms remain unexplored.

### Coregulators of bZIP28- and bZIP60-mediated gene regulation

TFs often work with coregulators at specific gene promoters to coordinate transcriptional changes dynamically, supporting various biological pathways (56). Multiple studies suggest that bZIP28 and bZIP60 operate alongside other transcriptional regulators: (a) protein–protein interaction analyses revealed interactions of bZIP28 and bZIP60 with other transcriptional regulators including TBP-ASSOCIATED FACTOR 12b (TAF12b) and NF-Y subunits; (b) multiple TF-binding motifs, enriched alongside ER stress–associated CREs, were found on promoters of genes differentially regulated by bZIP28 and/or bZIP60; and (c) chromatin immunoprecipitation sequencing (ChIP-seq) identified significant enrichment of TF-binding motifs on *in vivo* binding peaks of bZIP28 or bZIP60 (35, 46). The sequence of ERSE-I (5′-CCAAT-N_10-_CACG-3′) highlights how CCAAT-binding factors, also known as NF-Y subunits, modulate the transcriptional activity of UPR-bZIP TFs as activators. Although there are numerous NF-Y subunits (10 NF-YA, 10 NF-YB, and 10 NF-YC), the functional trimers and their related biological pathways remain largely unexplored (57). Given that bZIP TFs often form heterodimers with other bZIP TFs (58), and the second subunit of ERSE-I contains the core sequence of the G-box sequence (a known bZIP TF binding site), three bZIP TFs—ELONGATED HYPOCOTYL 5 (HY5) (59), G-class bZIP TF 2 (GBF2) (60), and ABA-Insensitive 5 (ABI5) (24)—have been identified and functionally characterized as coregulators of bZIP28 and bZIP60 through extensive experimental and computational approaches. Notably, HY5 and GBF2 act as repressors of UPR-bZIP TF activity, whereas ABI5 functions as an activator. Beyond TFs, non–DNA-binding transcriptional regulators also modulate the transcriptional activities of bZIP28 and bZIP60. For example, Nonexpressor of Pathogenesis-Related 1 (NPR1), a redox-regulated master regulator of salicylic acid (SA)–dependent responses (61), represses the transcriptional activities of bZIP28 and bZIP60 (62), whereas TAF12b, a key component of transcription preinitiation complexes (63), enhances the transcriptional activity of bZIP60 (64). These findings demonstrate that the transcriptional activities of bZIP28 and bZIP60 are finely tuned by a variety of coregulators, depending on the phase, type, and intensity of ER stress. However, the identification of coregulators involved in UPR-bZIP TF responses to ER stress induced by viral infection and the underlying mechanisms remains poorly understood, presenting a critical area for future research.

### Other regulators associated with ER stress–induced apoptosis in mammals, yeast, and plant PCD

While the UPR initiates prosurvival mechanisms to alleviate ER stress, it also triggers apoptosis or PCD when ER dysfunction cannot be resolved because of prolonged or severe stress. Thus, the initiation and regulation of ER stress–induced apoptosis are integral components of the UPR and are implicated in numerous critical cellular processes, including responses to viral infections across metazoans. Bax inhibitor 1 (BI-1) is a highly conserved ER-resident master regulator that can suppress BAX-mediated cell death in yeast and mammals (65). BI-1 primarily regulates ER stress–induced cell death by modulating the ER calcium levels and influencing IRE1α activity (66), although the mechanisms linking BI-1 to UPR regulators remain largely unexplored in both metazoans and plants (67). Interestingly, despite the absence of BAX homologs in plants, plant BI-1 can suppress BAX-mediated cell death in yeast (68).

A forward genetic screen aimed at identifying suppressors of *ire1a/b*, a *IRE1a* and *IRE1b* double mutant, lethality under ER stress led to the discovery of PIR1, an E3 ubiquitin ligase acting downstream of IRE1 (24). The missense mutation of *PIR1* suppress the lethality (*i.e.*, cell death) of *ire1a/b*, resulting in reduced sensitivity to prolonged ER stress. PIR1 also indirectly regulates the protein stability of ABI5, a transcriptional coregulator for bZIP28 and bZIP60, under ER stress, suggesting a role in balancing prosurvival and prodeath pathways. Another recent addition to the collection of UPR-related cell death regulators is BON-ASSOCIATED PROTEIN 2 (BAP2), which represses UPR biomarker genes while promoting prodeath mechanisms under conditions of UPR insufficiency (69). Taken together, these findings indicate that the UPR acts as a regulatory hub in which multiple sensors detect and relay stress signals, which are subsequently amplified by various regulators based on the progression of stress resolution, creating a highly interconnected signaling network. In the following section, we will discuss how the UPR responds to pathogen infections and functions in antiviral defenses.

## Molecular mechanisms of UPR-mediated antiviral defenses

RNA viruses that infect both mammals and plants are known to engage with the UPR and ERAD machinery to maintain ER homeostasis, creating a cellular environment conducive to infection. While much is understood about how mammalian viruses activate or suppress components of the UPR (for in-depth discussion, see other published reviews (14, 15)), significant progress is also being made in plant virology to unravel these critical virus–host interactions. Recent findings reveal that plant viral factors can modulate the UPR machinery by either activating or suppressing its signaling components and influencing ER stress–associated cell death regulators. These observations suggest that plant viral proteins possess a distinctive ability to activate multiple signaling pathways originating from the ER or modulate the crosstalk between the UPR and other stress responses (18, 21, 70). In this section, we provide recent insights into how certain plant viruses exploit UPR mechanisms to promote cell survival while enhancing their replication and movement. A deeper understanding of the convergence of these pathways is crucial for developing innovative crop breeding and antiviral strategies. Targeting multiple host factors, rather than focusing on individual components, could significantly improve the effectiveness of interventions against plant viruses.

### Overview of potexvirus and potyvirus interactions with the ER

**Figure 1:**
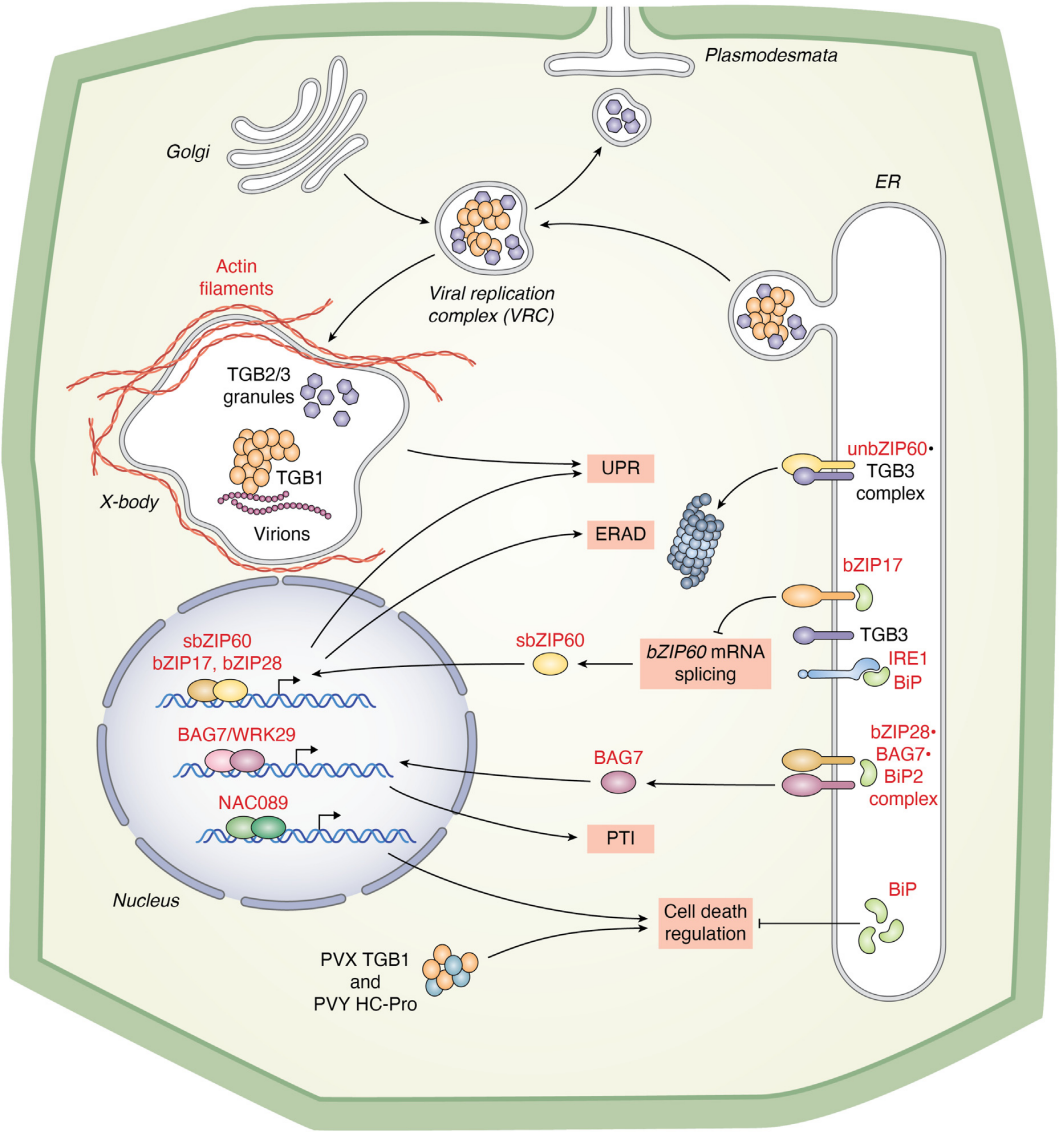
**Schematic representation of potexvirus interactions with plant UPR, ERAD, and cell death pathways.** VRCs, originating from the ER and Golgi, interact with plasmodesmata or aggregate into perinuclear X-bodies. TGB1 protein aggregates induce membrane rearrangements to form the core of X-body, which may cause ER stress and potentially trigger the UPR. Meanwhile, the viral TGB3 protein, an ER-associated protein, directly activates the IRE1-and bZIP60-mediated pathways. TGB3 interacts with unbZIP60, both of which are targeted for degradation *via* ERAD. In addition, BAG7 and NAC089 participate in pattern-triggered immunity signaling and the regulation of PCD. Other forms of PCD may be induced through the combinatorial actions of TGB1 and HC-Pro. The ER-resident chaperone BiPs play a critical role in maintaining ER homeostasis during ER stress and protecting plant cells from PCD. BiP, binding immunoglobulin protein; bzip60, basic leucine zipper 60; ER, endoplasmic reticulum; ERAD, ER-associated protein degradation; IRE1, inositol-requiring enzyme 1; PCD, programmed cell death; TGB1, triple gene block 1; unbZIP60, unspliced bZIP60; UPR, unfolded protein response; VRC, viral replication complex.

Two potexviruses, potato virus X (PVX) and plantago asiatica mosaic virus (PlAMV), exemplify the intricate virus–host interactions involving ER structure remodeling (Fig. 1) (71). These viruses induce ER membrane remodeling to form viral replication complexes (VRCs) and facilitate movement through plasmodesmata. Early in infection, viral movement proteins TGB2 and TGB3 form ER-derived granules that associate with TGB1 and VRCs near plasmodesmata, linking virus replication and cell-to-cell movement. At later stages, TGB2/3 granules aggregate into large perinuclear virus replication factories, known as X-bodies (72). TGB1, a multifunctional protein, acts as a silencing suppressor, modifies plasmodesmata, and organizes X-bodies by recruiting ER, Golgi membranes, and actin filaments to form structures containing viral RNA, virions, ribosomes, and granules (Fig. 1).

**Figure 2:**
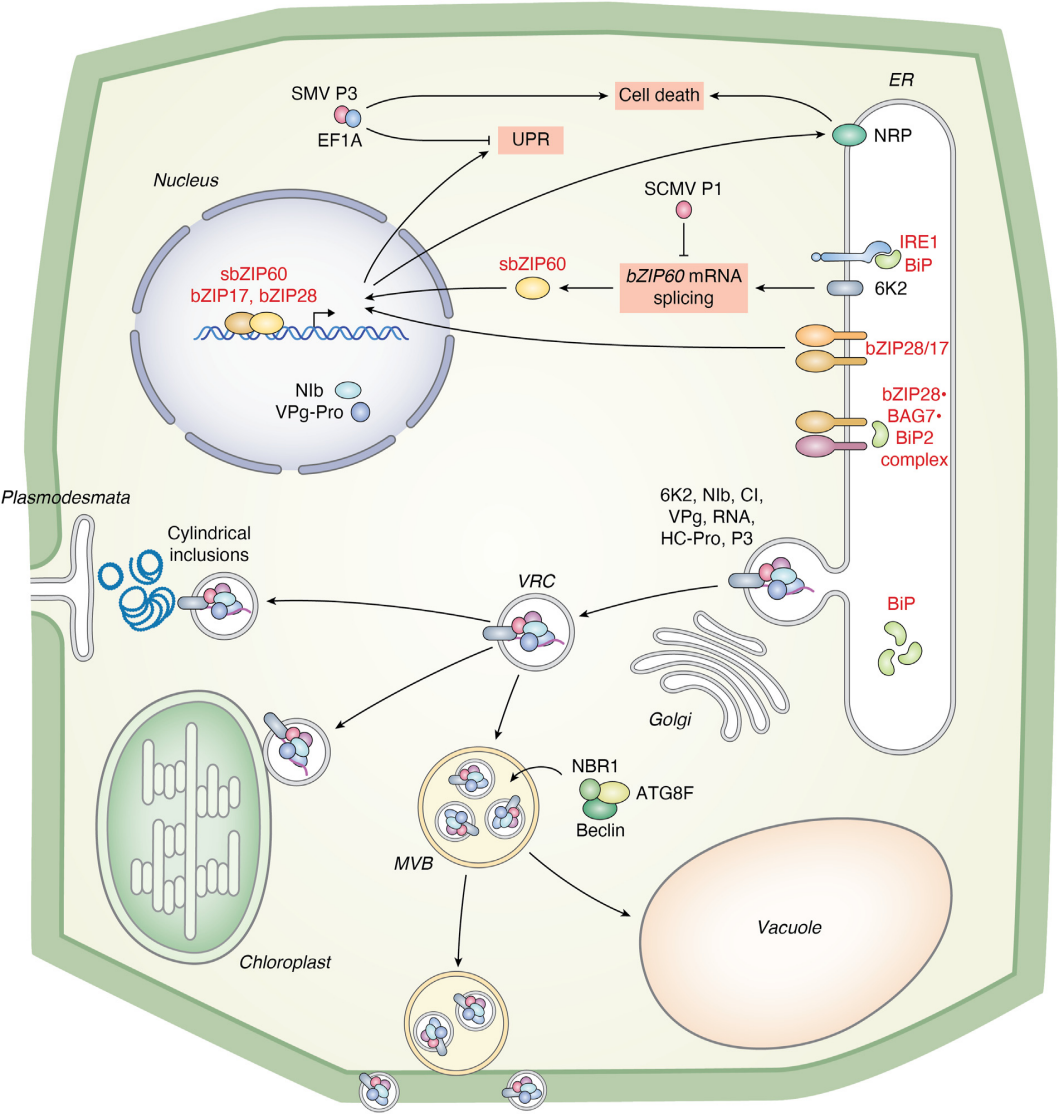
**Schematic representation of potyvirus interactions with plant UPR, autophagy, and cell death pathways.** This diagram illustrates the interactions between potyviruses and the UPR, highlighting the modulation of key cellular pathways, including autophagy, and cell death. SMV P3 interacts with cellular EF1A to inhibit the UPR and trigger cell death. SCMV P1 blocks *bZIP60* mRNA splicing, suppressing the IRE1-mediated UPR pathway. TuMV (turnip mosaic virus) and PVY (potato virus Y) 6K2 proteins activate both IRE1- and bZIP28-mediated UPR pathways. In addition, TuMV and other potyvirus 6K2 proteins are associated with the ER to form VRCs. Potyviral proteins NIb and VPg-Pro localize to the nucleus, where they are hypothesized to influence the transcriptional activities of bZIP60 and bZIP28. Mature VRCs bud off and merge with multivesicular bodies (MVBs), incorporating autophagy-related factors, such as NBR1, ATG8f, and Beclin-1, which promote viral replication. These autophagy receptors may also facilitate the turnover of viral replication proteins. In some cases, VRC–MVB complexes are transported to the cell wall, whereas others are directed to viral cylindrical inclusions and plasmodesmata to enhance cell-to-cell movement. Additional VRCs interact with chloroplasts. The interconnections between the UPR, autophagy, and cell death pathways underscore the complex strategies employed by potyviruses to manipulate host cellular processes during infection. bZIP28, basic leucine zipper 28; bZIP60, basic leucine zipper 60; EF1A, eukaryotic elongation factor 1A; SMV, soybean mosaic virus; TuMV, turnip mosaic virus; UPR, unfolded protein response; VRC, viral replication complex.

Potyvirus, members of the Potyviridae family, also rely on ER remodeling for replication and cell-to-cell movement (73, 74, 75, 76, 77). Although direct evidence linking potyvirus-induced membrane remodeling to UPR activation is limited, insights obtained for potexviruses (Fig. 1) suggest this as an avenue worth investigating. The potyvirus 6K2 protein generates convoluted membrane structures from the ER, which bud into vesicle-like VRCs containing viral and host proteins (*e.g.*, HC-Pro, P3, CI [cylindrical inclusion], 6K2, VPg, and Nib) (Fig. 2). In turnip mosaic virus (TuMV), 6K2 drives VRC assembly and membrane remodeling by interacting with host factors, such as the coatomer protein Sec24A, root hair defective 3 (RHD3), the ER SNARE Syp71, and the synaptotagmin SYTA (76, 78, 79, 80). These vesicles bypass the Golgi apparatus and travel to (i) plasmodesmata, directed by the CI protein, (ii) chloroplast membranes for continued replication, or (iii) multivesicular bodies, a prevacuolar compartment (81). Upon fusion with multivesicular bodies, VRCs are either degraded in vacuoles or transported to the cell periphery for RNA release (Fig. 2).

Virus-induced ER remodeling supports replication while triggering UPR activation because of altered ER membrane dynamics. Potexvirus–potyvirus synergism often results in spreading necrosis, linking to TGB1 and potyvirus HC-Pro activities (82). Some potexviruses, such as alternanthera mosaic virus and bamboo mosaic virus, target chloroplast or mitochondrial membrane for VRC assembly, employing Golgi and autophagic vesicles for adding scaffolding (83, 84, 85, 86). However, their ER remodeling patterns differ from PlAMV and PVX (83, 85). These differences underscore the diversity in how plant viruses modulate the UPR through membrane remodeling.

### Modulation of the UPR by plant viral proteins

In addition to triggering membrane remodeling, several plant viruses encode proteins that directly modulate key steps in the UPR during infections, such as autophagy and ERAD. Among these are viral transmembrane proteins that elicit ER-to-nucleus signaling *via* IRE1, bZIP28, and/or bZIP17. Notable viral proteins include TGB3 from garlic mosaic virus, PlAMV, and PVX; TGB1 from PVX; 6K2 from TuMV and potato virus Y; P5 from lettuce infectious yellows crinivirus; P9 from rice black-streak dwarf virus; and a beta-satellite encoded protein C1 (βC1) associated with tomato yellow leaf curl China virus (16, 20, 21, 87, 88, 89, 90). TGB1 differentially modulates the bZIP60 suppressor (82).

TGB3 from PVX and PlAMV, which possesses the N-terminal transmembrane domain, inserts into the ER membrane, activating IRE1, bZIP28, and bZIP17 to varying extents (Fig. 1). Although the mechanism of TGB3-induced IRE1 activation remains unclear, studies reveal that unspliced *bZIP60* (*unbZIP60*) transcripts persist, whereas sbZIP60 functions as a nuclear TF (16, 17). Intriguingly, *bZIP17* knockout increases *sbZIP60* mRNA levels and PVX accumulation, suggesting bZIP17 may regulate IRE1-mediated *bZIP60* splicing and influence virus accumulation. Further complexity arises as unbZIP60, an ER-resident transmembrane protein, interacts with TGB3 along the ER membrane (21), although its function remains poorly understood. This interaction suggests that TGB3 turnover, potentially regulated by unbZIP60 and bZIP17, may modulate the TGB3-induced UPR during infection. Future research is needed to determine whether bZIP17 exclusively regulates IRE1-mediated *bZIP60* splicing or if it also impacts the IRE1-dependent mRNA decay in support of PVX or PlAMV infection.

The small transmembrane protein 6K2 of TuMV, a known VRC marker and UPR inducer (13, 91), primarily regulates *bZIP60*, *bZIP28*, and the selective autophagy receptor gene *NBR1* (19, 88). NBR1 interacts with the TuMV RNA-dependent RNA polymerase NIb, and the autophagy protein ATG8f, forming a complex that colocalizes with 6K2-labeled VRCs (Fig. 2). Overexpression of *NBR1* or *ATG8f* enhances TuMV replication, whereas deficiencies hinder infection. In addition, ATG8f interacts with tonoplast intrinsic protein 1 (TIP1) that encases NBR1–ATG8f-containing VRCs at the tonoplast. Observation of membrane-bound viral particles in TuMV-infected cell vacuoles suggests that bZIP28 and bZIP60 may promote the expression of autophagy-related genes, including *NBR1* and *ATG8f*, which virus hijack to form VRCs (19).

The UPR appears to play virus-specific roles as highlighted by the double knockdown of *bZIP60* and *bZIP17*, which increases PlAMV coat protein levels and viral titers in both inoculated and systemic leaves (16). This aligns with findings that silencing *bZIP60* enhances the susceptibility of *Nicotiana benthamiana*, a model host, to infection by the nonhost pathogen *Pseudomonas cichorii* (92). Notably, TGB3 induces the expression of UPR-related genes encoding BiPs, calreticulin, and calmodulin, and SKP1, a key component of the SCF-type E3 ubiquitin ligase complex involved in ERAD (18, 93, 94). While bZIP60's downstream targets, including *NAC103* and *NAC089*, have been characterized, bZIP17's transcriptional activities remain less understood. Overexpression of *NAC103* inhibits plant growth and alters leaf morphology, potentially reflecting growth or developmental changes associated with viral infection (16). NAC089, linked to PCD (52), connects the UPR to cellular stress responses during infection. Overexpression of *TGB3* can also trigger oxidative stress and necrosis, alleviated by BiPs, suggesting that *TGB3* overexpression induces ER stress and oxidative damage, or that UPR activation leads to NAC103-mediated PCD (Fig. 1).

### UPR-mediated cell death regulation during viral infections

Overexpression of potexvirus *TGB3* from a heterologous virus genome can trigger PCD, likely because of cytotoxic ER stress. Studies have shown that TGB3-induced cell death can be mitigated by overexpressing BiPs, underscoring BiPs' critical role in alleviating ER stress–induced PCD (18, 95). Interestingly, other potexviral proteins, such as the PVX TGB1 protein, synergistically induced PCD when coexpressed with the potato virus Y HC-Pro, despite not being embedded in the ER. This coexpression not only triggers cell death but also leads to ER fragmentation and oxidative stress (Fig. 1). TGB1 and HC-Pro coexpression further activates bZIP60-mediated transcription and enhances ERAD to remove misfolded proteins from the ER. BiPs serve as a crucial modulator, regulating these stress responses and preventing excessive cell death (82).

BAG7, initially identified in studies linking pathogen-induced PCD and the UPR (54), plays a multifaceted role in viral infection. BAG7 binds BiP2 in the ER, delaying cell death (54). In *bag7* knockout plants, BiP levels are elevated, leading to increased virus accumulation, although virus-induced cell death is unaffected (16, 54, 96). This highlights BAG7's importance in modulating ER stress during infection. BAG7's function extends beyond the ER, as it relocates to the nucleus upon dissociation from BiP. In the nucleus, BAG7 acts as a cofactor with WRKY29 to drive transcriptional changes associated with pattern-triggered immunity) (96, 97). It remains unclear whether BAG7's interactions with BiP and WRKY29 reflect distinct proviral and antiviral roles in different subcellular compartments or whether these functions converge to limit ER stress–induced cell death.

Arabidopsis BI-1, a well-characterized inhibitor of PCD, resides in the ER and interacts with enzymes related to very-long-chain fatty acid synthesis (VLCFA), such as cytochrome b5 (AtCb5) and ECERIFERUM 10 (CER10), for plant sphingolipid biosynthesis, contributing to the synthesis of 2-hydroxylated VLCFAs in response to oxidative stress (98). Emerging evidence suggests that physical changes in ER structure, possibly mediated by BI-1, might be sensed by the UPR machinery. Although research linking potexvirus or potyviral infection to VLCFA or lipid biosynthesis is limited, studies on tombusviruses reveal that VRCs convert cellular membranes into large viral replication organelles by hijacking lipid synthesis and modification pathways (99, 100). Intriguingly, *BI-1* knockdown leads to increased levels of PlAMV and TuMV without necrosis (17), suggesting a potential role in regulating sphingolipid metabolism during viral infection. This raises the possibility that plant viruses reprogram lipid metabolism for their benefit, although further research is needed to elucidate BI-1's role in ER stress and viral pathogenesis.

In soybean (*Glycine max* L.), eukaryotic elongation factor 1A (EF1A) has been identified as a key regulator of ER stress–induced PCD. The soybean mosaic virus (SMV), a potyvirus, encodes the P3 protein, which associates with the ER and induces UPR-related transcriptional changes (101). A yeast two-hybrid screen using a soybean expression library identified the GmEF1A protein as a P3-interaction protein that negatively regulates antiviral defenses and cell death. Knockdown of *EF1A* in soybean increases virus accumulation while impairing the plants' ability to respond to ER stress (101), highlighting its dual role in antiviral restriction and stress response.

Taken together, the UPR functionally intersects viral infections at multiple molecular levels, forming a complex signaling network. Current models, such as the interactions of the UPR with potexvirus (Fig. 1) and potyvirus (Fig. 2) infections, provide valuable insights into the regulation, mechanisms, and functional implications of these processes. Further research into these interactions will enhance our understanding of UPR-mediated responses and their potential for developing novel strategies to mitigate virus-induced stress in plants.

## Systems-level understanding of the UPR in response to viral infection

The intricate coordination of UPR signaling pathways by ER stress sensors, UPR-bZIP TFs, and their coregulators makes it extremely challenging to elucidate the underlying molecular and genetic mechanisms. To address this challenge, the plant UPR research community has adopted systems-level approaches to explore key biological questions, including the functional relationship between the UPR and viral infection responses. Particularly, in the postgenomics era, numerous omics datasets specifically associated with plant UPR have been generated, providing a foundational resource for understanding the functional role of the UPR in response to viral infection. In this section, we introduce how omics data and resulting biological networks have provided evidence of the critical role played by the UPR in linking molecular and physiological responses to viral infection. We also discuss the potential involvement of plant hormones in the signaling crosstalk. For a more in-depth discussion of omics approaches for understanding the UPR, we recommend reading other published reviews and research articles (23, 102).

### Omics approaches unveiling the functional link between the UPR and viral infection

Transcriptome profiling has been successfully applied over the past decades to investigate the transcriptomic landscape in response to ER stress, elucidate the roles of key UPR regulators, and discover new regulatory elements (35, 36, 38, 48). Although these studies did not specifically aim to understand the UPR–pathogen infection link, they have provided crucial insights. For instance, Mishiba *et al.* conducted microarray analysis in Arabidopsis WT and *ire1a/b* under ER stress treated with Actinomycin D, a transcriptional inhibitor, to identify RIDD targets (38). The analysis revealed that genes upregulated in *ire1a/b*, but not WT, under ER stress were highly enriched for stress-related Gene Ontology terms, with “defense response” being the top-ranked (49 of 190 genes, including *PATHOGENESIS-RELATED 4* [*PR4*]). This indicates that many RIDD target genes are also responsive to viral infection. Similarly, RNA-sequencing (RNA-seq) analysis in maize during persistent ER stress, conducted by Srivastava *et al.*, demonstrated that ER stress triggers broad transcriptomic alterations, including hundreds of genes peaking specifically in the middle and late phases, with significant enrichment of “responses to biotic stimulus” and “responses to stress, defense” (48). In addition to transcriptome analyses, TF-DNA interactome studies have provided mechanistic insights into UPR gene regulation related to viral infection. Enhanced yeast one-hybrid analyses in maize (103) and Arabidopsis (60) tested the binding affinity of 2,000 Arabidopsis and 500 maize TFs against selected UPR biomarker genes, identifying 10 Arabidopsis and 14 maize WRKY TFs that are closely associated with viral infection responses and bind to the promoters of *bZIP60*, respectively, suggesting that WRKY TFs may directly coordinate *bZIP60* expression across plant species. The time-course ChIP-seq analysis of bZIP28 and bZIP60 in Arabidopsis during ER stress recovery discovered substantial overlap in the binding targets of both bZIP28 and bZIP60, with strong enrichment for ER stress–related pathways, as expected (35). Notably, bZIP28-specific binding targets showed significant enrichment in biotic stress–related pathways, suggesting a direct role for bZIP28 in regulating responses to viral infection. Collectively, these global findings demonstrate that the UPR is highly interconnected with regulators, signaling components, and metabolic genes responsive to viral infection, showcasing the utility of omics approaches in revealing the molecular mechanisms underlying the UPR in response to viral infection.

### Networks built on omics datasets revealing the UPR–viral infection axis

While the omics datasets described previously serve as valuable resources for investigating the functional link between the UPR and viral infection—as well as other biological pathways—additional steps are required for the effective extraction of biological insights. Constructing biological networks based on omics data and analyzing network properties (*e.g.*, predicting regulatory hub genes) have been invaluable in exploring complex signaling pathways, such as the UPR. Through RNA-seq analysis during recovery from ER stress, Ko and Brandizzi (35) identified multiple coexpression modules of ER stress–responsive genes regulated by bZIP28 and/or bZIP60, which are highly enriched for biotic stress–related pathways, suggesting that viral infection–responsive transcriptional networks also function in growth re-establishment during ER stress recovery. Furthermore, the Arabidopsis enhanced yeast one-hybrid study mentioned previously constructed a highly interconnected TF network map based on the TF-DNA interactome, revealing that defense-related pathways are exclusively enriched in TFs binding to *bZIP60* promoters but not to the other gene promoters (60). This suggests that TFs upstream of bZIP60 may be responsive to viral infection. Thus, while further investigation is needed with innovative experimental and computational approaches, systems-level analyses have significantly advanced our understanding of how the UPR functionally links to viral infection responses within interconnected networks.

### Crosstalk among plant hormone signaling pathways and their influences on UPR gene regulation

**Figure 3:**
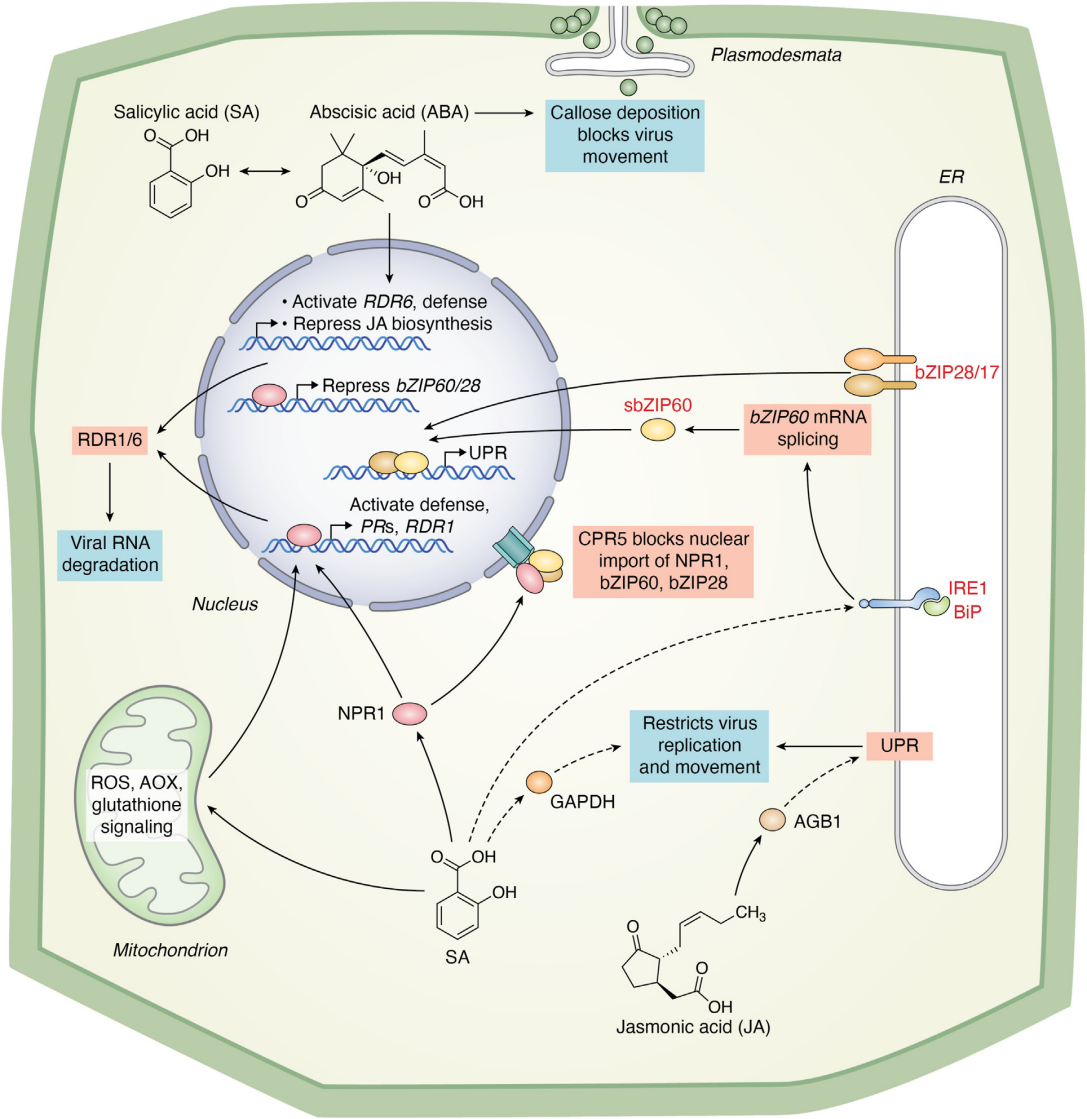
**Hormonal regulation of the UPR by ABA, JA, and SA.** This diagram illustrates how ABA, JA, and SA can directly or indirectly modulate the UPR. SA, through the NPR1 pathway, activates antiviral defense genes, including *RDR1*, which, alongside *RDR6*, degrades viral RNA. In addition, SA likely limits viral replication and movement through the glycolysis enzyme GAPDH. SA also influences ROS production in mitochondria and retrograde signaling *via* AOX and glutathione. ABA induces the expression of *RDR1* and *RDR6*, linking it to antiviral silencing, and further represses the JA signaling pathway while limiting viral movement by promoting callose deposition at plasmodesmata. CPR5 plays a crucial role in balancing plant growth and ER stress response by utilizing the UPR to repress the bZIP28 and IRE1–bZIP60 pathways. ABA, abscisic acid; AOX, alternative oxidase; bZIP28, basic leucine zipper 28; bZIP60, basic leucine zipper 60; ER, endoplasmic reticulum; IRE1, inositol-requiring enzyme 1; JA, jasmonic acid; NPR1, Nonexpressor of Pathogenesis-Related 1; RDR1, RNA-dependent RNA polymerase 1; ROS, reactive oxygen species; SA, salicylic acid; UPR, unfolded protein response.

SA is the first identified plant signaling molecule known to trigger resistance against a wide range of pathogens, including viruses (104, 105). SA occupies a central hub in the plant defense regulatory network (106). Exogenous SA application acts through branched pathways to inhibit viral replication, cell-to-cell movement, and systemic spread. Decades of research on SA-induced antiviral resistance continue to uncover new SA-associated factors and mechanisms that directly influence infection (106). Here, we propose that the UPR and ERAD pathways represent additional mechanisms by which SA restricts virus replication and movement (Fig. 3).

SA induces the expression of genes encoding pathogenesis-related proteins, most of which, including NPR1, contribute directly to innate immunity and indirectly to limiting virus infection (107). An exception is NPR1's involvement in PlAMV resistance in Arabidopsis. SA drives reactive oxygen species accumulation in mitochondria, retrograde signaling through alternative oxidase, and glutathione production, inducing resistance to PVX, cucumber mosaic virus, and tobacco mosaic virus (105, 108) (Fig. 3). Through NPR1, SA induces the expression of *RNA-dependent RNA polymerase 1* (*RDR1*), encoding a key player in the RNA silencing pathway that limits viruses in meristematic tissues (109). Host RDRs amplify RNA silencing signals by synthesizing double-stranded RNAs, which are processed by Dicer-like enzymes into siRNAs. These siRNAs guide homologous viral genomes and mRNA degradation, thereby restricting infection. Notably, RDR6, another silencing component, is co-induced by abscisic acid (ABA), a stress-inducible plant hormone, thereby linking ABA to antiviral RNA silencing. ABA also regulates callose deposition at plasmodesmata, limiting virus movement between cells. The interplay between SA and ABA, often described as antagonistic during abiotic stress responses, converges in the regulation of jasmonic acid (JA) biosynthesis and plasmodesmata defenses (Fig. 3). Interestingly, while JA promotes antiviral defenses in rice, ABA represses JA biosynthetic genes.

SA has long been implicated as an ER stress signaling molecule, as it induces the transcription of UPR biomarker and promotes the splicing of *bZIP60* (110). Until recently, most models of SA-mediated antiviral defenses overlooked the link between SA, the UPR, and viral infection. Since the UPR restricts potyvirus and potexviral infections, it is reasonable to consider that the UPR serves as another arm of SA-led antiviral defenses. Two important mechanistic insights into SA-mediated UPR signaling are noteworthy: (i) it predominantly relies on IRE1a, not IRE1b, and SA treatment can compensate for immunity deficiencies in *IRE1a* knockout mutants; and (ii) it operates through NPR1, which does not regulate the expression of *bZIP60* (110) but represses transcriptional activities of bZIP28 and bZIP60 *via* direct interaction in an SA-dependent manner (62). CONSTITUTIVE EXPRESSER OF PATHOGENESIS-RELATED GENES 5 (CPR5), a negative regulator of SA responses, also balances growth and ER stress responses by repressing bZIP28- and IRE1-bZIP60-mediated pathways (111). These findings highlight the complex interplay between UPR regulators and SA signaling components, warranting extensive genetic, genomic, and systems-level investigation for deeper understanding.

A recent study by Ko and Brandizzi (60) revealed a significant overlap between ABA-responsive genes and those reprogrammed during ER stress and recovery. Notably, ABA levels did not increase under ER stress in both WT and *ire1a/b* mutants (24), suggesting that certain ABA-responsive genes may have multifunctional roles in the UPR that are independent of ABA signaling.

Guanine nucleotide-binding proteins (G proteins), consisting of Gα, Gβ, and Gγ subunits, are also implicated in plant defense responses through interactions with hormones such as JA (112). The single Gβ subunit in Arabidopsis, GTP-binding protein B1 (AGB1), does not affect sensitivity to ER stress when knocked out individually; its loss enhances growth defects of *ire1a/b* (113). This suggests a synergistic role for AGB1 and IRE1 in the ER stress response (Fig. 3). Moreover, the synergy extends to viral infection, as bacterial growth rates in *agb1-2* *ire1a/b* triple mutants were significantly higher than in either single mutant. Taken together, these findings underscore the intricate roles of plant hormones in mediating UPR responses during viral infection, offering a new framework for exploring their regulatory networks.

## Conclusions and perspectives

Here, we discuss the functional link between the UPR and responses to viral infections, highlighting recent findings and providing insights into the potential of systems-level approaches to better understand the full landscape. Emerging evidence suggests that the UPR responds to viral infections through a highly interconnected network of sensor proteins, transcriptional regulators, downstream effectors, and their dynamic interactions to determine cell fate. Future studies should aim to dissect the complexity of the UPR-plant immune network at higher spatial and temporal resolution. New technological advances could make this challenging mission feasible. For example, combining functional genomics tools with machine learning–based network algorithms could help establish temporal gene regulatory networks underlying UPR-bZIP TF activities against viral infections and predict regulatory hubs, providing a dynamic view of the UPR and possibly revealing multiphasic regulation (114, 115). Single-cell and spatial genomics technologies could further help explore the dynamic expression of key downstream or regulatory genes in a cell type–specific manner, capturing details that may be lost in bulk cell approaches (116). Finally, as demonstrated in responses to other environmental stresses (6, 31, 51, 117, 118), the UPR may play a central regulatory role, receiving viral infection signals and relaying them to numerous biological pathways associated with growth, development, and fitness. Exploring these areas using traditional forward genetic approaches will be crucial to identifying downstream factors and characterizing the underlying mechanisms. We hope that the strategies outlined here will prove fruitful in advancing this pursuit.

## Supporting information

This article contains supporting information.

## Conflict of interest

The authors declare that they have no conflicts of interest with the contents of this article.

## Supporting information

Checklist
