# Supplementary material for: ER stress and viral defense: Advances and future perspectives on plant unfolded protein response in pathogenesis
Source: J Biol Chem. 2025 Feb 25;301(4):108354. doi: 10.1016/j.jbc.2025.108354 (PMC11982459; doi:10.1016/j.jbc.2025.108354)
Supplement: Checklist [file mmc1.pdf]

Data presentation check list:

This checklist is intended to help authors submit manuscripts that present data and figures in a transparent manner that is compatible with JBC guidelines and best practices in the field.

1. **Have you reviewed the JBC instructions to authors and guidelines on data presentation?**
   - [x] Yes
   - [ ] No *(Review available resources using the links above)*
2. **Does your manuscript contain any grayscale or color images (gels, blots, micrographs of cells or tissues)?**
   - [ ] Yes
   - [x] No (*Please skip to question 3*)

   a) Were adjustments in brightness or contrast applied equally to the entire image?
   - [ ] Yes
   - [ ] No
   - [ ] Not Applicable

   b) Were there any non-linear adjustments (gamma) applied to the images?
   - [ ] Yes
   - [ ] No

   c) Were any of the images digitally manipulated ("beautified") to obscure or remove any bands, debris or background?
   - [ ] Yes
   - [ ] No

   d) Are all the unmodified original data (complete gels or fields) available on demand for review?
   - [ ] Yes
   - [ ] No

   e) For images of gels/blots: (*Skip to Q.2f if not applicable*)
   Click "Blot Images and Quantification" button on JBC guidelines web page for information.

   i) Were the positions of any splices made to remove irrelevant lanes from the gel images clearly indicated in the figures?
   - [ ] Yes
   - [ ] No
   - [ ] Not Applicable

   ii) Do the methods clearly describe how gel/blot images were developed and captured?
   - [ ] Yes
   - [ ] No

   iii) Are molecular weight markers above and below the band(s) of interest indicated?
   - [ ] Yes
   - [ ] No

   If no, please provide justification (with space for text box)

   iv) Do the methods define how the specificity of each antibody used was validated?
   - [ ] Yes
   - [ ] No
   - [ ] Not applicable

JBC JOURNAL OF BIOLOGICAL CHEMISTRY

v) Are immunoblots for post-translational modifications (PTMs) (phosphorylation, methylation, etc) accompanied by parallel blots for total protein levels?

☐ Yes
☐ No
☐ Not applicable

If no, please provide justification (with space for text box)

vi) Are the gels/blots quantitatively analyzed?

☐ Yes
☐ No (Please skip to question 2f)

- Do the methods indicate how signals were quantified?

☐ Yes
☐ No

- Are signals normalized to the total protein loaded to each lane?

☐ Yes
☐ No

If no, please provide justification (with space for text box)

- Are PTM signals normalized to the total level of the protein of interest?

☐ Yes
☐ No

If no, please provide justification (with space for text box)

f) For micrographs of cells/tissue: (Skip to Q.3 if not applicable)
Click "Image Data" button on JBC guidelines web page for information.

i) Do the methods define how the specificity of each antibody used was validated?

☐ Yes
☐ No
☐ Not applicable

ii) Is a scale bar shown in the image and defined in the figure legend?

☐ Yes
☐ No

iii) Are images depicting a single color shown in gray scale (...for the color blind)?

☐ Yes
☐ No
☐ Not applicable

iv) Are any colors used selected to be accessible to the color blind?

☐ Yes
☐ No
☐ Not applicable

Click here AND here for guidance on preparing images for the color blind

ASBMB JOURNALS

3. Do any of the figures contain bar graphs?

- ☐ Yes
- ☑ No *(Please skip to question 5)*

Click "Quantitative Data and Statistics" button on JBC guidelines web page for information.

a) Are the results plotted as the mean ± S.D.

- ☐ Yes
- ☐ No

If no, please provide justification

b) Are individual data points super-imposed on the bar graph?

- ☐ Yes
- ☐ No

i. If yes, does the figure legend explicitly define individual data points as technical replicates (i.e., assay duplicates/triplicates, etc) or independent biological replicates?

- ☐ Yes
- ☐ No

If no, please provide justification

4. Do the conclusions depend on comparisons between multiple quantitative measurements?

- ☐ Yes
- ☐ No *(Please skip to question 5)*

Click "Quantitative Data and Statistics" button on JBC guidelines web page for information.

a) Is the statistical significance of differences between results/groups evaluated?

- ☐ Yes
- ☐ No *(Please skip to question 5)*

b) Are data tested for a normal distribution?

- ☐ Yes
- ☐ No *(Please skip to question 4c)*

i) Do the methods describe how?

- ☐ Yes
- ☐ No

c) Are any data sets tested for statistical outliers?

- ☐ Yes
- ☐ No *(Please skip to question 4d)*

i) Do the methods describe how?

- ☐ Yes
- ☐ No

d) Are the statistical tests used in each panel specifically defined in each figure legend?

- ☐ Yes
- ☐ No

e) Is the specific type of t test (paired/unpaired; 1/2-tailed, one sample) defined for each use?

- ☐ Yes
- ☐ No
- ☐ Not applicable

f) Are multiple data sets compared in any figure panel?

- ☐ Yes
- ☐ *No (Please skip to question 5)*

i) Is a 1-way or 2-way ANOVA used as needed for the number of variables?

- ☐ Yes
- ☐ No

ii) Does each figure legend report the full results of the ANOVA?

- ☐ Yes
- ☐ No

iii) Does each figure legend identify the post hoc testing method used?

- ☐ Yes
- ☐ No

iv) Are exact p values indicated in the figure or figure legend?

- ☐ Yes
- ☐ No

5. Were any of the experiments performed in cell lines?

- ☐ Yes
- ☑ No *(Please skip the following questions)*

a) Do you describe the source/origin of cell line(s)?

- ☐ Yes
- ☐ No

b) Do you indicate whether and how the authenticity of the cell line(s) was validated (using short tandem repeat (STR) profiling or other methodology)?

- ☐ Yes
- ☐ No

c) Do you suppress the expression of specific endogenous target genes in the cell line(s)?

- ☐ Yes
- ☐ No *(Please skip the following questions)*

Click "Gene Expression and Genetic Manipulation" button on JBC guidelines web page for information.

i. Do you use sh/siRNAs to manipulate gene expression?

- ☐ Yes
- ☐ No *(Please skip to question 5.c.ii)*

- Do you provide the sequences of the sh/siRNAs used?
  - ☐ Yes
  - ☐ No

- Please indicate the controls used by checking the appropriate boxes. Do you:
  - ☐ Use two si/shRNAs targeting different portions of the target gene?
  - ☐ Use control siRNAs with sequences altered from the target?
  - ☐ Verify depletion of the targeted protein?
  - ☐ Functionally rescue effects of knockdown by re-expressing the target gene?

ii. Do you use CRISPR to target a specific endogenous gene?

☐ Yes

☐ No *(Please skip the following questions)*

- Do you provide the sequences of all guide RNAs?
  - ☐ Yes
  - ☐ No
- Please indicate the controls used by checking the appropriate boxes. Do you:
  - ☐ Use two or more clones of the manipulated cell lines?
  - ☐ Use two or more guide RNAs targeting different portions of the target gene?
  - ☐ Use control isogenic cells that retain a wild-type allele?
  - ☐ Verify depletion (or mutation) of the targeted protein?
  - ☐ Functionally rescue effects of the manipulation by re-expressing the target gene?
